# Chicken DDX1 Acts as an RNA Sensor to Mediate IFN-β Signaling Pathway Activation in Antiviral Innate Immunity

**DOI:** 10.3389/fimmu.2021.742074

**Published:** 2021-09-23

**Authors:** Zhenyu Lin, Jie Wang, Wenxian Zhu, Xiangyu Yu, Zhaofei Wang, Jingjiao Ma, Hengan Wang, Yaxian Yan, Jianhe Sun, Yuqiang Cheng

**Affiliations:** School of Agriculture and Biology, Shanghai Jiao Tong University, Shanghai Key Laboratory of Veterinary Biotechnology, Agriculture Ministry Key Laboratory of Urban Agriculture (South), Shanghai, China

**Keywords:** chicken, dsRNA sensor, DDX1, IRF7, IFN-β

## Abstract

Chickens are the natural host of Newcastle disease virus (NDV) and avian influenza virus (AIV). The discovery that the RIG-I gene, the primary RNA virus pattern recognition receptor (PRR) in mammals, is naturally absent in chickens has directed attention to studies of chicken RNA PRRs and their functions in antiviral immune responses. Here, we identified Asp-Glu-Ala-Asp (DEAD)-box helicase 1 (DDX1) as an essential RNA virus PRR in chickens and investigated its functions in anti-RNA viral infections. The chDDX1 gene was cloned, and cross-species sequence alignment and phylogenetic tree analyses revealed high conservation of DDX1 among vertebrates. A quantitative RT-PCR showed that chDDX1 mRNA are widely expressed in different tissues in healthy chickens. In addition, chDDX1 was significantly upregulated after infection with AIV, NDV, or GFP-expressing vesicular stomatitis virus (VSV-GFP). Overexpression of chDDX1 in DF-1 cells induced the expression of IFN-β, IFN-stimulated genes (ISGs), and proinflammatory cytokines; it also inhibited NDV and VSV replications. The knockdown of chDDX1 increased the viral yield of NDV and VSV and decreased the production of IFN-β, which was induced by RNA analog polyinosinic-polycytidylic acid (poly[I:C]), by AIV, and by NDV. We used a chicken IRF7 (chIRF7) knockout DF-1 cell line in a series of experiments to demonstrate that chDDX1 activates IFN signaling *via* the chIRF7 pathway. Finally, an *in-vitro* pulldown assay showed a strong and direct interaction between poly(I:C) and the chDDX1 protein, indicating that chDDX1 may act as an RNA PRR during IFN activation. In brief, our results suggest that chDDX1 is an important mediator of IFN-β and is involved in RNA- and RNA virus-mediated chDDX1-IRF7-IFN-β signaling pathways.

## Introduction

The first line of defense against an invasion of pathogenic microorganisms is innate immunity, and this system is evolutionarily conserved in different organisms. The innate immune system quickly recognizes exogenous insults such as viruses and bacterium; the system then induces a series of immune mechanisms to resist the infection, including an interferon-mediated antiviral response and an interleukin-mediated pro-inflammatory response (1). The innate immune system is particularly critical for detecting invading pathogens and activating subsequent adaptive immunity, and the type I IFN response is an effective defense against viral infections (2, 3). The production of a type I IFN relies on the host’s pattern recognition receptors (PRRs), which recognize and bind to specific pathogen-associated molecular patterns (PAMPs) and then activate related receptor-dependent signaling cascades to induce IFN production (4).

PAMPs are evolutionarily conserved molecular structures in pathogens that are vital to themselves, and PRRs are receptors in the host that can recognize specific PAMPs to activate the immune system; they include toll-like receptors (TLRs), nucleotide binding oligomerization domain (NOD)-like receptors (NLRs), and retinoic acid-inducible gene-I (RIG-I) -like helicases (RLHs) (5–8). RLHs, including RIG-I, LGP2, and MDA5, are part of the DExD/H-box helicase family and have been demonstrated to be extremely important antiviral PRRs (9). For most cells, RLHs can detect non-self nucleic acid components in the cytoplasm. All RLHs have a DExD/H-box RNA helicase domain and a C-terminal domain (CTD), which is responsible for recognizing and binding to viral RNA (10). Interestingly, RIG-I, an important PRR involved in sensing viral RNA, is absent in chickens (11).

Recent studies have shown that several DExD/H-box helicases that are not in the RIG-I family are involved in recognizing viruses and/or conducting the downstream signaling axis (12). The DExD/H-box RNA helicases are named for an Asp-Glu-Ala-Asp (DEAD) or Asp-Glu-Ala-His (DEAH) motif within the helicase domains (13). The DExD/H-box RNA helicases are a group of proteins that are conserved in the evolution of multiple species, including at least eight conserved motifs. They are involved in ATP binding, ATP hydrolysis, nucleic acid binding, and helicase activity (14, 15). They have been proven to play a key role in all aspects of RNA metabolism; their functions in virus replication, cell cycles, somatic reprogramming, autophagy, and cancer development are gradually becoming clear. Here, we explore their roles in the innate immune system (16–18).

DHX9 and DHX36 are MyD88-dependent DNA sensors in plasmacytoid dendritic cells that are involved in the host’s antiviral cytokine response. DHX9 is critical to the activation of NF-κB and to the production of TNF-α and IL-6 (19). The RNA sensor DHX33 can interact with NLRP3 to form an inflammasome complex after stimulation by a dsRNA virus, leading to the secretion of IL-18 and IL-1β (20). DDX50 can combine with RIG-I and MDA5 to inhibit the replication of dengue virus 2 by upregulating the production of IFN-β (21). The DNA sensor DDX41 directly binds DNA and STING *via* its DEAD domain to exert functions through the STING-TBK1-IRF3 pathway (22), DDX3 and DDX60 promote RIG-I binding to viral RNA to trigger the type I IFN signal axis (23). Similarly, DHX29 binds directly to nucleic acids and interacts with MAVS and RIG-I through the helicase 1 domain, activating the RIG-I-MAVS-dependent signaling pathways (24). In addition to these positive regulatory effects in innate immunity, some members of DExD/H-box RNA helicases, such as DDX6 and DDX24, play a negative regulatory role in antiviral responses. DDX6 can be hijacked by viruses to inhibit the activation of immune genes. DDX24 may weaken RLR signaling by competing with RIG-I for RNA and disrupting the interaction between IRF7 and the receptor interacting protein 1; this interaction is essential to activating innate immune genes (25, 26).

DDX1 is a member of the DEAD box helicase family and was originally identified from a retinoblastoma cell line (27). There are two sides to the role of DDX1 in innate immunity. On the one hand, DDX1 can upregulate the expression of type I IFN and inhibit virus replication. For example, DDX1 inhibits FMDV replication; this is dependent on ATPase/helicase activity. It also simultaneously activates the expression of IFN in infected cells (28). DDX1 binds to the nsp14 protein of TGEV and induces the host’s innate immune response (29). On the other hand, DDX1 can also be hijacked by some viruses and promote virus proliferation. For example, DDX1 interacts with the nonstructural protein 14 (nsp14) of coronavirus infectious bronchitis virus (IBV) and severe acute respiratory syndrome coronavirus (SARS-CoV) to promote virus replication (30). The non-structural protein 3 (nsp3) of Venezuelan equine encephalitis virus (VEEV) interacts with DDX1 and DDX3 in infected U87MG glioblastoma cells to facilitate viral multiplication of the U87MG glioblastoma cell to promote virus replication (31).

The innate immune systems of chickens and mammals are quite different. Chickens have a smaller repertoire of immune genes than mammals. Many key immune genes involved in innate immunity, such as TLR8, TLR9, Riplet, and IRF9, are missing in chicken cells (32). Especially the discoveries that both the DExD/H-box helicases in mammals RIG-I and DHX9 are naturally absent in chickens has directed attention to studies of chicken DExD/H-box helicases and their functions in antiviral immune responses (11, 33). In our previous studies, we have reported that the chicken DDX41 and DDX3X participates in anti-DNA and -RNA virus innate immunity, respectively (34, 35). In a preliminary experimental study, we found that expression of chicken DDX1 increased significantly in DF-1 cells after infection with RNA viruses. This indicated that chDDX1 may play a role in immune regulation of chickens after RNA virus infections. Here, we tried to verify its functions in antiviral innate immunity and explored the mechanisms.

In the present study, chDDX1 was cloned from DF-1 cells, and its role in the type I IFN signaling pathway was explored. The results showed that chDDX1 can bind to poly(I:C), a simulant of dsRNA, and rely on IRF7 to activate IFN-β to inhibit virus replication. In brief, our results suggest that chDDX1 is an important IFN-β mediator that is involved in RNA- and RNA virus-mediated chDDX1-IRF7-IFN-β signaling pathways. These findings expand existing knowledge of the relationship between the innate immunity of birds and mammals and contribute to our understanding of the biological role of DDX1 in the evolution of innate immunity.

## Materials and Methods

### Cells and Viruses

DF-1 is a chicken embryonic fibroblast cell line from East Lansing strain eggs. The chIRF7 gene knockout DF-1 cell line (IRF7^−/−^) was obtained through the CRISPR/Cas9 technique, as described in our previous study (36). Chicken embryo fibroblast cells (CEFs) were prepared from 10-day-old specific-pathogen-free (SPF) embryos which were obtained from Merial Vital (Beijing, China). The CEFs, DF-1 and IRF7^−/−^ DF-1 cells were maintained in high-glucose Dulbecco’s modified Eagle’s medium (DMEM) (Gibco, USA) containing 10% fetal bovine serum (FBS) (Gibco) and 1% penicillin-streptomycin (Gibco). All cells were incubated in 5% CO_2_ at 37°C. The A/chicken/Shanghai/010/2008 (H9N2) virus (SH010), a H9N2 subtype of AIV, was isolated from chickens in Shanghai, China, in 2008. The NDV strain NSD14 was isolated from chickens in a farm of Shandong province, China. Green fluorescent protein (GFP) tagged NDV low virulent strain LaSota named NDV-GFP, and GFP tagged vesicular stomatitis virus (VSV) VSV-GFP, were stored in our laboratory. These viruses were purified, propagated, and stored as described in our previous study (37).

### Cloning and Bioinformatics Analysis of chDDX1

Based on the chDDX1 sequence (NM_204563.1) obtained from the National Center for Biotechnology Information (NCBI), the primers chDDX1-F and chDDX1-R (**Table 1**) located outside the chDDX1 open reading frame (ORF) were designed and used to amplify chDDX1 cDNA *via* RT-PCR from DF-1 cells. The PCR product was ligated into a pTOPO-Blunt vector (Aidlab, Beijing, China) for sequencing, and the positive colonies were sent to the Beijing Genomics Institute (Beijing, China) for sequencing. The amino acid sequence of chDDX1 was aligned with the other animal DDX1 proteins from humans, mice, pigs, and ducks using Clustal W and edited with ESPript 3.0 (http://http://espript.ibcp.fr/ESPript/cgi-bin/ESPript.cgi). Sequence homology and a phylogenetic analysis of the DDX1 amino acid sequences were conducted using DNASTAR. A phylogenetic tree was constructed based on the DDX1 from 18 different species, including mammals, birds, and fish. Different domains in the chDDX1 amino acid sequences were predicted using the simple modular architecture research tool (SMART) program (http://smart.embl-heidelberg.de/). Homology modeling for chDDX1 was conducted using the online protein-modeling server Swiss-Model (http://swissmodel.expasy.org/).

**Table 1:** PCR primers used in the study.

Target gene	Purpose	Name	Sequence of oligonucleotide (5’–3’)
**NDV**	qRT-PCR	NDV F	TGCAGCAATGGTACTCCGTT
		NDV R	CCTTTGCTACCGTGACCCAT
**chIFN-α**	qRT-PCR	qIFN-α F	AACCACCCACGACATCCTTC
		qIFN-α R	TGTCGCTGCTGTCCAAGC
**chIFN-β**	qRT-PCR	qIFN-β F	CCTCAACCAGATCCAGCATT
		qIFN-β R	GGATGAGGCTGTGAGAGGAG
**chIFN-λ**	qRT-PCR	qIFN-λ F	CCTGGCCTTCCTTACCCAAG
		qIFN-λ R	CTCAGTTTCCCAGAGGGCTG
**chIRF-7**	qRT-PCR	qIRF-7 F	GCCTGAAGAAGTGCAAGGTC
		qIRF-7 R	CTCTGTGCAAAACACCCTGA
**chPKR**	qRT-PCR	qPKR F	TGCTTGACTGGAAAGGCTACT
		qPKR R	TCAGTCAAGAATAAACCATGTGTG
**chMx-1**	qRT-PCR	qMx-1 F	GTTTCGGACATGGGGAGTAA
		qMx-1 R	GCATACGATTTCTTCAACTTTGG
**IL-6**	qRT-PCR	qIL-6 F	GACGAGGAGAAATGCCTGAC
		qIL-6 R	GACTTCAGATTGGCGAGGAG
**IL-8**	qRT-PCR	qIL-8 F	GCTCTGTCGCAAGGTAGGAC
		qIL-8 R	GGCCATAAGTGCCTTTACGA
**chβ-actin**	qRT-PCR	qβ-actin F	CAGACATCAGGGTGTGATGG
		qβ-actin R	TCAGGGGCTACTCTCAGCTC
**chDDX1**	qRT-PCR	qDDX1 F	CGACAAACTCTGGGAAAGGC
		qDDX1 R	TTGTGTTCCTTGATTGCCCG
	To obtain sequence	chDDX1 F	ATGGCGGCGTTCTCGGAAAT
		chDDX1 R	TCAGAATGTTCTGAACAGCT
	Construction of chDDX1	p3×FLAG-14-CMV *Kpn* I F	gatagatctgatatcggtaccATGGCGGCGTTCTCGGAA
		p3×FLAG-14-CMV *Bam*H I R	tttgtagtcagcccgggatccGAATGTTCTGAACAGCTGGTTAGG
	Construct truncated forms of chDDX1	d SPRY U	AGTCACACCTCTAGTTGC
		d SPRY L	caactagaggtgtgactTTCAAATTTCCACCAAAAGATGG
		d HELICc U	ACAATCTATTTTTGTCCTGCAG
		d HELICc L	gacaaaaatagattgtAGGATGGGTCTGGCAATTTC
		d 1-100 aa U	GGTACCGATATCAGATCTATC
		d 1-100 aa L	gatatcggtaccatgGCTTTTGCAATTGGATC
		d 1-250 aa U	GGTACCGATATCAGATCTATC
		d 1-250 aa L	gatatcggtaccatgCCAAAAGATGGCTATATTGG
		d 1-500 aa U	GGTACCGATATCAGATCTATC
		d 1-500 aa L	gatatcggtaccatgATCAAGGAACACAAGATGG
		d 251-500 aa U	TGGAAATTTGAAGTCTTCTTCAC
		d 251-500 aa L	gaagacttcaaatttccaATCAAGGAACACAAGATG
		d 501-740 aa U	TGCCCGAACAGTGTACTC
		d 501-740 aa L	tacactgttcgggcaGGATCCCGGGCTGACTACAAAG

### Plasmid Construction

FLAG-tagged chDDX1 plasmids were constructed by inserting full-length chDDX1 into the *Kpn* I and *Bam*H I sites p3 × FLAG-14-CMV of the expression vector using a ClonExpress II one-step cloning kit (Vazyme, Nanjing, China). The primers used in the PCR are listed in **Table 1**. The truncated plasmids of chDDX1, including dSPRY domain, dHELICc domain, d1-100 aa (deletion of 1-100 amino acids), d1-250 aa, d1-500 aa, d251-500aa, and d501-740 aa, were constructed using a modified homologous recombination method and the primers listed in **Table 1**. The chIFN-β promoter luciferase reporter plasmids (pGL-chIFN-β-Luc), containing -158 to +14 of the chicken IFN-β promotor motif, were constructed as described in our previous study (37).

### Luciferase Reporter Assay

The CEFs, DF-1 cells and IRF7^−/−^ DF-1 cells were plated in 24-well plates at 5 × 10^5^/0.5 mL and were transiently transfected with the reporter plasmid pGL-chIFN-β-Luc (120 ng/well) and the control Renilla luciferase (pRL-TK, 60 ng/well); the cells were also transfected with the indicated plasmids using Nulen PlusTrans™ Transfection Reagent (Nulen, Shanghai, China). The cells were lysed 24 hours after transfection, and luciferase activity was detected using a Dual-Luciferase Reporter Assay System kit (Promega, USA) according to the manufacturer’s instructions. Renilla luciferase activity was used for normalization. All reporter assays were repeated at least three times.

### RNA Extraction and Quantitative Real-Time PCR

In order to detect the expression of DDX1 mRNA in different chicken tissues, tissues from the brain, trachea, lungs, and 13 other types of tissues were collected from three specific pathogen free (SPF) chickens (Merial Vital Laboratory Animal Technology Company, China) at four weeks of age. All animal experiments were conducted in accordance with the animal welfare standards approved by the Ethical Committee for Animal Experiments of Shanghai Jiao Tong University, China. After the RNA was extracted from the chicken tissues or cells using an HP Total RNA kit (Omega, USA), the RNA was reverse transcribed to cDNA using a cDNA synthesis kit (Vazyme). The viral RNA in the supernatant is extracted by a TaKaRa MiniBEST Viral RNA/DNA Extraction Kit (TaKaRa, Japan). Quantitative real-time PCR (qRT-PCR) tests were conducted using the primers listed in **Table 1** and an ABI 7500 RT-PCR system. The qRT-PCR was performed according to the manufacturer’s instructions using a ChamQ™ SYBR^®^ qPCR Master Mix (Vazyme). The conditions and data processing of the qRT-PCR were the same as those in our previous study (38).

### Expression of Short Hairpin RNA

Specific chDDX1 gene knockdown in DF-1 cells was conducted *via* pGPU6-based silencing (GenePharma, Shanghai, China). A negative control sequence that is not present in human or chicken genome databases and two RNA interference (RNAi) target sequences against chDDX1 were designed (**Table 2**). The complementary short hairpin RNA (shRNA) template oligonucleotides were synthesized based on the RNAi sequences designed against chDDX1 and the negative control. Next, the complementary oligonucleotides were annealed and inserted into the shRNA expression vector pGPU6-neo. The recombinant shRNA plasmids were called shDDX1-1, shDDX1-2, and shNC, respectively. For silencing, the DF-1 cells were plated in 12-well or 24-well plates and transfected with shRNA at 1 µg/well or 0.5 µg/well using Nulen PlusTrans™ Transfection Reagent (Nulen). Forty-eight hours after transfection, the cells were used for the subsequent experiments.

**Table 2:** RNAi target sequences.

Target gene	Name	Sequence of oligonucleotide (5’–3’)	Accession No.
**chDDX1**	shDDX1-1	GCTTTCAGCATTCCGGTTATC	NM_204563.1
	shDDX1-2	GCAACTAGAGGTGTGACTAAA	
**Negative control**	shNC	GTTCTCCGAACGTGTCACGT	

### Western Blot Analysis

The DF-1 cells were plated in 6-well plates at 2 × 10^6^/2 mL and then transfected with a total of 4 µg empty plasmid or various expression plasmids. Thirty-six hours later, the transfected cells were washed twice with phosphate buffer saline (PBS) (Gibco) and then lysed with a cell lysis buffer (Beyotime, Shanghai, China) containing a protease cocktail (Yeasen, Shanghai, China) and phenylmethylsulfonyl fluoride (PMSF) (Yeasen). The lysate was centrifuged at 13,000 rpm for 15 minutes to obtain the supernatant, and a 5 × SDS loading buffer was added before the lysates were boiled for ten minutes. The proteins isolated from the cell lysates were separated *via* SDS-PAGE and analyzed using western blotting. Images were obtained using the Tanon 5200 imaging system (Tanon, Shanghai, China), as described in our previous study (35).

### *In-Vitro* Poly(I:C) Pulldown Assays

The DF-1 cells were plated in 6-well plates and transfected with various plasmids. The transfected cells were harvested 24 hours after transfection and lysed in the same way as before the western blot analysis. The poly(I) and agarose beads coupled with poly(C) were resuspended in a buffer containing 50 mM Tris (pH = 7) and 150 mM NaCl to a final concentration of 2 mg/mL and mixed at a ratio of 2 to 1 by volume. The mixture was incubated at 56°C for 30 minutes; the temperature was then slowly reduced to 4°C to obtain poly(I:C)-coated beads. The poly(C) and poly(I:C) coupled agarose beads were incubated with cell lysates at 4°C for six hours on a low-speed rotating instrument (15 rpm/min). After the mixture was centrifuged at 6000 rpm for one minute, the supernatant was discarded, and the beads were washed three times with TBS. A loading buffer of 60 µL 1 × SDS was added to the beads, and this mixture was boiled for ten minutes and then centrifuged under the same conditions as the supernatant for the western blot analysis.

### Virus Infection and Poly(I:C) Stimulation

The DF-1 cells were plated, washed twice with PBS, and infected at 0.1 multiplicity of infection (MOI) with AIV, NDV, or VSV-GFP. The RNA from the cells, which was infected with the viruses at different times, was then collected for qRT-PCR. Poly(I:C) (Invivogen, San Diego, CA) was transfected into the DF-1 cells at 0.05 µg/well (in 24-well plates), and the cells were used for the subsequent experiments 12 hours after transfection.

### Statistical Analysis

The data were expressed as means ± standard deviations. Significance was determined using a two-tailed independent Student’s t test (*p < 0.05, **p < 0.01).

## Results

### Cloning and Sequence Analysis of chDDX1

**Figure 1:**
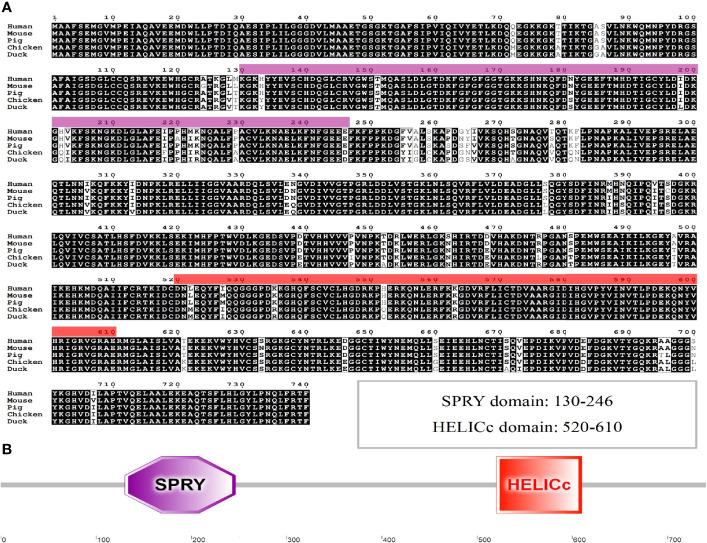
**(A)** Alignment of the amino acid sequence of chDDX1 with chDDX1 proteins from humans (NP_004930.1), mice (NP_598801.1), pigs (ANR02618.1) and ducks (XP_027311055.1). The amino acid sequences of different animals were aligned using Clustal W and edited with ESPript 3.0. The black shading indicates amino acid identity, and the gray shading indicates similarity (50% threshold). **(B)** Protein domains of chDDX1 predicted by SMART.

To better understand the role of chDDX1 in innate immunity in chickens, chDDX1 was cloned and studied using bioinformatics analysis. The open reading frame of chDDX1 contains 2223 bp and encodes 741 amino acid residues. The DDX1 amino acid sequences in humans (*Homo sapiens*, NP_004930.1), mice (*Mus musculus*, NP_598801.1), pigs (*Sus scrofa*, ANR02618.1), and ducks (*Anas platyrhynchos*, XP_027311055.1) are 93.2%, 93.1%, 93.5%, and 98.6% identical to those in chickens (*Gallus gallus*, NP_989894.1), respectively (**Figure 1A**). The protein domains of chDDX1 were predicted using SMART. The results show that chDDX1 consists of an N-terminal domain in SPla and the RYanodine receptor (SPRY) (aa 130-246) and a C-terminal helicase superfamily c-terminal (HELICc) domain (aa 520-610) (**Figure 1B**).

### Phylogenetic Tree Analyses and the Three-Dimensional Structure of chDDX1

**Figure 2:**
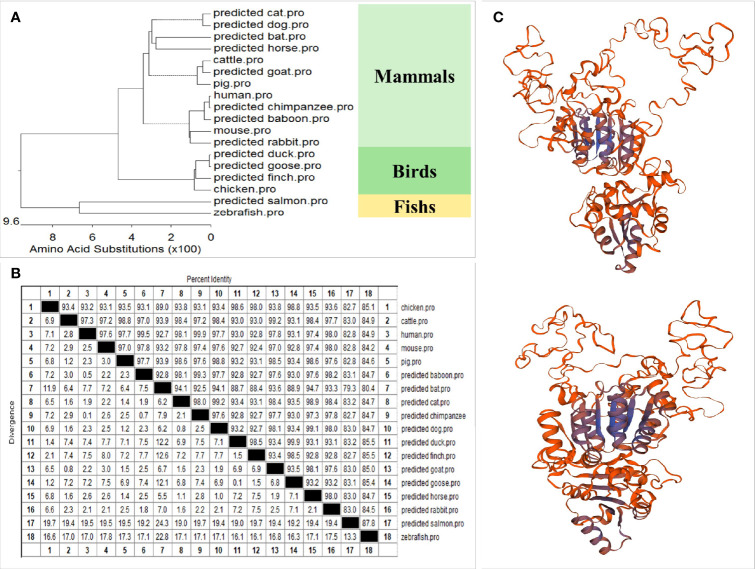
**(A)** Phylogenetic tree of vertebrate DDX1. A neighbor-joining phylogenetic tree of vertebrate DDX1 was generated with MegAlign software using DDX1 sequences from the following animals: chickens (NP_989894.1_1), cattle (NP_001068936.1), humans (NP_004930.1), mice (NP_598801.1), pigs (ANR02618.1), baboons (predicted, XP_021780285.1), bats (predicted, XP_023384316.1), cats (predicted, XP_011279621.1), chimpanzees (predicted, XP_001162067.2), dogs (predicted, XP_848865.1), ducks (predicted, XP_027311055.1), finches (predicted, XP_021381054.1), goats (predicted, XP_017911108.1), geese (predicted, XP_013028107.1), horses (predicted, XP_001502097.3), rabbits (predicted, XP_002710081.1), salmon (predicted, XP_014061742.1), and zebrafish (NP_001184253.1). **(B)** The amino acid sequence homology of different animals. **(C)** Three-dimensional structure of chDDX1 predicted by Swiss-Mode.

A phylogenetic tree was developed based on multiple alignments of DDX1 from various species, including fish, birds, and mammals (**Figure 2A**). The resulting phylogenetic tree consists of three major branches. The DDX1 protein sequences of chicken, finches (predicted), geese (predicted) and ducks (predicted) belong to one subgroup. The DDX1 of mammals, including humans, mice, rabbits (predicted), chimpanzees (predicted), baboons (predicted), cattle, horses (predicted), goats (predicted), dogs (predicted), pigs, bats (predicted), and cats (predicted) belong to another subgroup. Fish DDX1 sequences from zebrafish and salmon (predicted) belong to a third subgroup. These results reflect the genetic relationships among these species. The amino acid sequence homologies of different animals were conducted using MegAlign; the results are shown in **Figure 2B**. The predicted three-dimensional structures of chDDX1 are shown in **Figure 2C**.

### Tissue Distribution of chDDX1 Expression

**Figure 3:**
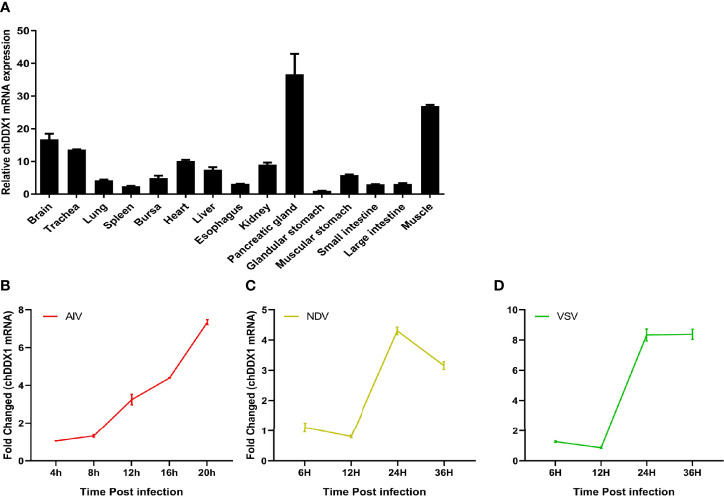
**(A)** Tissue distribution of chDDX1 mRNA in SPF chicken tissues. chDDX1 mRNA levels are expressed as relative mRNA indexes, calculated as the index (the copy number of chDDX1 mRNA/the copy number of β-actin mRNA) of the test tissue divided by the index of muscle. **(B)** Upregulation of chicken DDX1 in DF-1 cells infected with AIV at 0.1 MOI. **(C)** Upregulation of chDDX1 in DF-1 cells infected with NDV at 0.1 MOI. **(D)** Upregulation of chDDX1 in DF-1 cells infected with VSV-GFP at 0.1 MOI.

The chicken tissues tested included brain, trachea, lung, spleen, bursa, heart, liver, esophagus, kidney, pancreatic gland, stomach (glandular and muscular stomach), intestine (small and large intestine), and muscle. The results showed that chDDX1 mRNA was widely expressed in most tissues analyzed. The highest levels were observed in the pancreatic gland; higher levels were detected in muscle, brain, trachea, heart, liver, and kidney tissue; moderate levels in the muscular stomach, bursa, lungs, spleen, esophagus, and small and large intestine; and low levels in the glandular stomach (**Figure 3A**).

### Upregulation of chDDX1 Expression in Response to Viral Infection

In mammals, DDX1 is involved in the type I IFN-mediated antiviral innate immune response. However, the role of chDDX1 in the antiviral response remains unknown. For the host, upregulating the expression of certain immune-related genes is an important strategy in infection resistance. To determine whether chDDX1 could induce an antiviral response to infection with AIV, NDV, or VSV-GFP, we conducted a preliminary analysis of the expression of chDDX1 in DF-1 cells following infection with SH010 AIV, NSD14 NDV, and VSV-GFP. The results showed that the mRNA levels of chDDX1 were significantly upregulated during the early stages of viral infection (**Figures 3B–D**).

### chDDX1 Involved in the Regulation of IFNs, Pro-Inflammatory Cytokines, and ISGs; DDX1 Inhibits Viral Yield in Chickens

**Figure 4:**
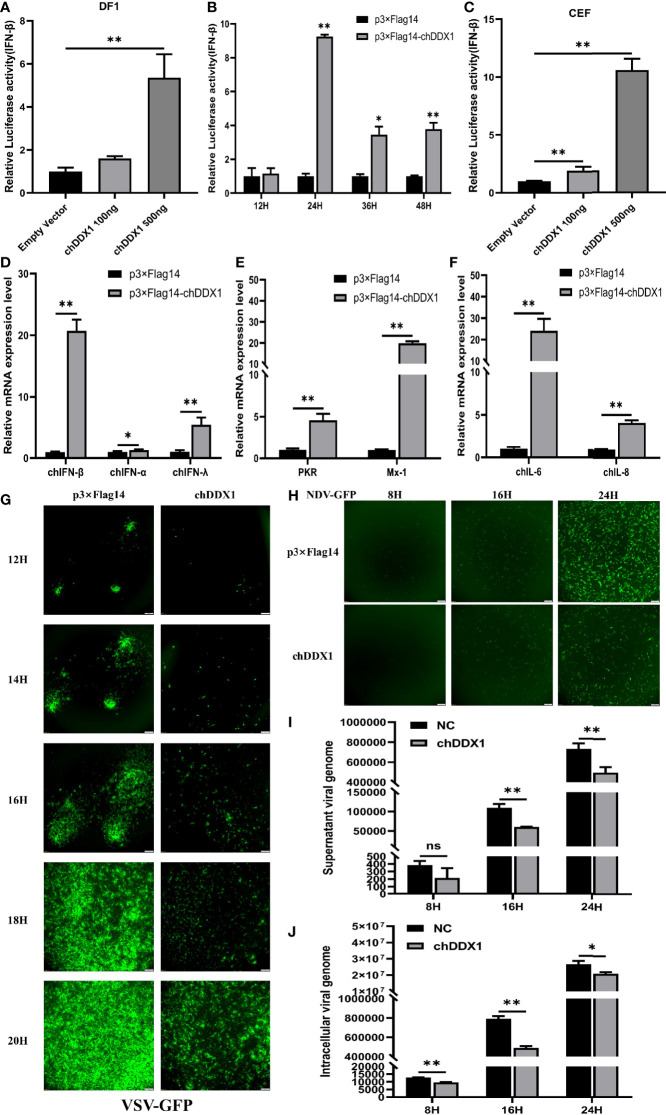
Chicken DDX1 were involved in the regulation of IFNs, pro-inflammatory cytokines, and ISGs and also inhibited viral yield. **(A)** DF-1 cells were co-transfected with luciferase reporter plasmids (pRL-TK and pGL-chIFN-β-Luc) and p3×FLAG-14-chDDX1 or p3×FLAG-14. Luciferase assays were performed after 24 hours of co-transfection. **(B)** chIFN-β luciferase activity at indicated time points. **(C)** CEFs were co-transfected with luciferase reporter plasmids and p3×FLAG-14-chDDX1 or p3×FLAG-14. Luciferase assays were performed after 24 hours of co-transfection. **(D)** Endogenous chIFNs mRNA after transfected with chDDX1 or an empty vector. **(E)** Relative mRNA levels of ISGs (PKR and Mx-1) after transfection with chDDX1 or an empty vector. **(F)** Relative mRNA levels of proinflammatory cytokines (IL-6 and IL-8) after transfection with chDDX1 or an empty vector. **(G)** Viral fluorescence in DF-1 cells transfected with p3×FLAG-14-chDDX1 or p3×FLAG-14 and infected with VSV-GFP at 0.1 MOI. Error bars represent standard deviations. **(H)** Viral fluorescence in DF-1 cells transfected with p3×FLAG-14-chDDX1 or p3×FLAG-14 and infected with NDV-GFP at 0.1 MOI. **(I)** NSD14 NDV virus genome detected by qRT-PCR in the supernatant of chDDX1-overexpressing DF1 cells at 8, 16 and 24 hours after infection (MOI, 0.1 for 8, 16 and 24 hours). **(J)** NSD14 NDV virus genome detected by qRT-PCR in chDDX1-overexpressing DF1 cells (MOI, 0.1 for 8, 16 and 24 hours). The difference between the experimental and control groups was *p < 0.05 or **p < 0.01.

DDX1 has been reported to activate type I IFN signaling in mouse dendritic cells (39). To investigate whether chDDX1 is also involved in the regulation of IFNs production, DF-1 cells, an immortalized chicken embryonic fibroblast cell line, were cotransfected with chDDX1 expression plasmids and with chIFN-β luciferase reporter plasmids. We found that overexpression of chDDX1 in DF-1 cells activated the IFN-β promoter, and this activation correlated positively with the dosage of the transfected chDDX1 plasmids (**Figure 4A**). Twenty-four hours after transfection, chDDX1 had the strongest activation effect on chIFN-β (**Figure 4B**). To further confirm the ability of IFN activation of chDDX1, we prepared primary chick embryo fibroblasts (CEFs). And luciferase assays were conducted with CEFs. Similarly, the overexpression of chDDX1 in CEFs activated the IFN-β promoter in a dose-dependent manner (**Figure 4C**).

To further study the effect of chDDX1 on the expression of innate immune genes, qRT-PCR was conducted after chDDX1 or empty vector transfected into DF1 cells. Consistent with the luciferase assay, the expression of chIFN-β mRNA was significantly upregulated by chDDX1 transfection (**Figure 4D**). Besides IFN-β, the mRNA levels of IFN-α, IFN-λ, and the IFN-stimulated genes (ISGs) (PKR and Mx-1) were also upregulated by chDDX1 transfection (**Figures 4D, E**). It is noted that the proinflammatory cytokines (IL-6 and IL-8) were also upregulated in chDDX1 transfected DF1 cells (**Figure 4F**).

Next, the chDDX1-overexpressing and normal DF-1 cells were infected with VSV-GFP and NDV-GFP, respectively, and fluorescence was measured with a fluorescence microscope. The fluorescence intensities of both VSV-GFP and NDV-GFP in chDDX1 overexpression cells were significantly lower than that in the control DF-1 cells at all tested time points (**Figures 4G, H**). This result suggests that chDDX1 overexpression in DF-1 cells suppresses VSV-GFP and NDV-GFP viral replication. Then, the chDDX1-overexpressing and normal DF-1 cells were further infected with an unmodified moderate virulent NDV strain NSD14, and the viral RNAs inside the cells and in the supernatants were detected by qPCR, respectively. Results showed that both the viral loads inside the cells and in the supernatants were significantly lower in chDDX1 overexpressing cells than those in the empty vector transfected groups (**Figures 4I, J**). In summary, the overexpression of chDDX1 in DF-1 cells induced the expression of IFNs, ISGs, and proinflammatory cytokines and also inhibited VSV-GFP and NDV replications.

### Essential Domains of chDDX1 in chIFN Activation

**Figure 5:**
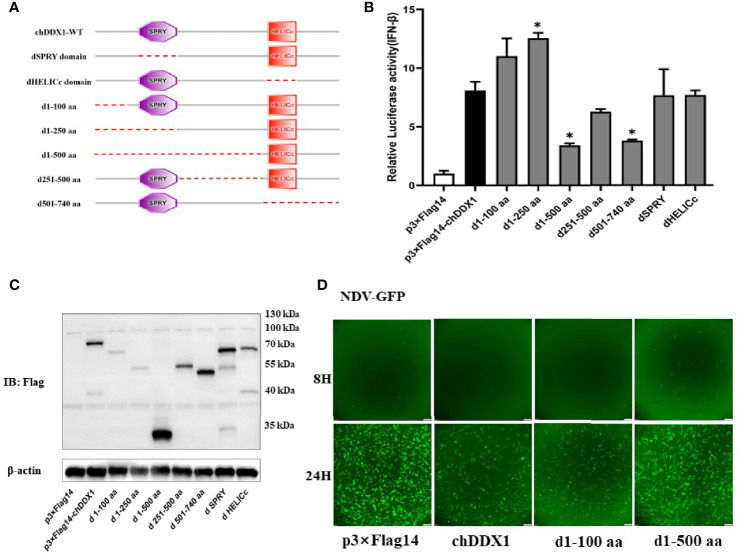
Essential domains of chDDX1 for IFN-β promoter activity. **(A)** Schematic structure of chDDX1 mutants. **(B)** The effects of chDDX1 truncated mutants on IFN-β promoter activity. **(C)** Western blots for expression of the truncated chDDX1 protein. **(D)** NDV-GFP Virus Proliferation in DF-1 cells transfected with p3×FLAG-14, p3×FLAG-14-chDDX1, chDDX1-d1-100, and chDDX1-d1-500 plasmids (MOI at 0.1 for 8 and 24 hours). The difference between the experimental and control groups was *p < 0.05.

Based on the secondary structure of chDDX1 predicted by the SMART program, a series of truncated mutants of chDDX1 were generated. The ability of these mutants to activate the IFN-β promoter was measured and compared to that of wild-type chDDX1 with dual luciferase reporter assays (**Figures 5A, B**). As shown in **Figure 5B**, the deletion of 100 (d1-100 aa) or 250 (d1-250 aa) amino acids (aa) at the chDDX1 N-terminal slightly increased IFN-β promoter activity. The further deletion of 500 residues in the chDDX1 N-terminal (d1-500 aa) resulted a significant decrease of promoter activity. The C-terminal deletion mutant (d501-740 aa) and the mutant deleted 251-500 amino acids (d251-500 aa) also led to a weaker ability to activate IFN-β promoter. However, deletion of the SPRY domain (dSPRY) or the HELICc domain (dHELICc) from chDDX1 did not significantly affect its ability to activate the IFN-β promoter. The expressions of the truncated chDDX1 proteins were detected using western blot, and all the protein bands were consistent with their indicated sizes (**Figure 5C**). These results indicate that the first 250 amino acids of chDDX1 is possible a negative regulatory domain for chDDX1’s IFN activation, and the 250-740 aa of chDDX1 contains pivotal domains that required for activating IFN-β signaling. To further verify the IFN activation abilities and the antiviral functions of the truncations, the DF1 cells transfected p3×FLAG, p3×FLAG-chDDX1, chDDX1-d1-100aa and chDDX1-d1-500aa were infected with NDV-GFP and fluorescence was measured. Consistently with the result of the IFN activation in the **Figure 5B**, the chDDX1-d1-100aa transfected cells showed a slight stronger inhibitory effects, while the chDDX1-d1-500aa cells exhibited an obvious weaker inhibitory effects on the NDV-GFP replication, when compared with the wild type chDDX1 transfected cells (**Figure 5D**).

### Knockdown of chDDX1 Decreased IFN-β Production and Increased Viral Yield

**Figure 6:**
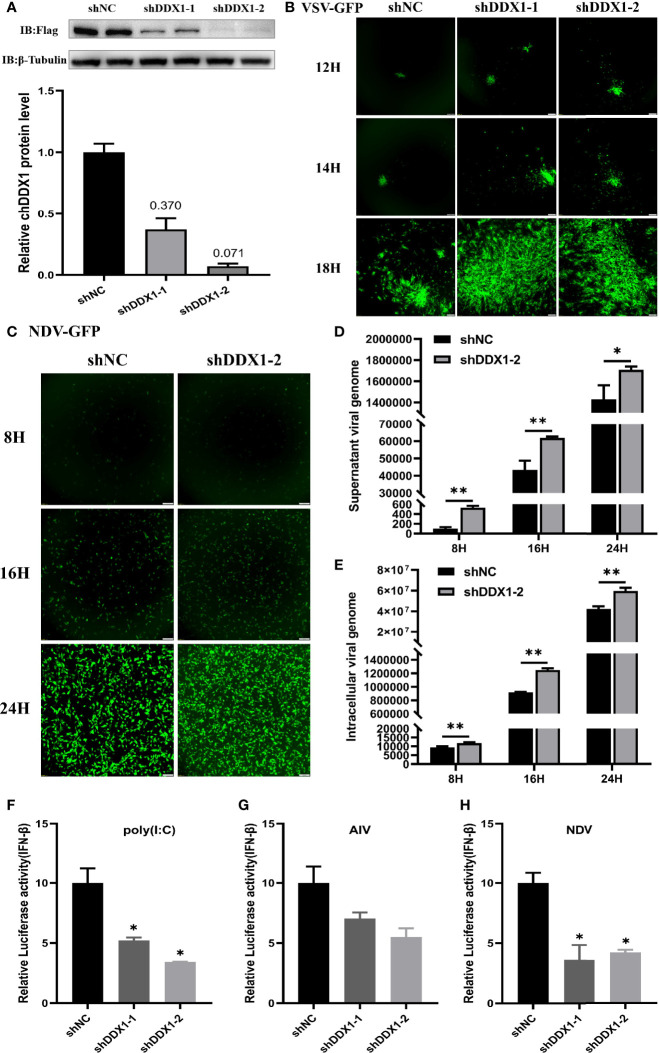
chDDX1 knockdown decreased IFN-β production and increased viral yield. **(A)** Protein levels of chDDX1 in DF-1 cells cotransfected with expressing plasmid p3×FLAG-14-chDDX1 and RNAi plasmids (shDDX1-1, shDDX1-2, or shNC). Expression levels of FLAG-tagged chDDX1 were quantified using western blotting, and immunodetection was measured using anti-FLAG Ab. **(B)** Virus fluorescence in DF-1 cells transfected with RNAi plasmids of chDDX1 and infected with VSV-GFP (MOI = 0.1). **(C)** Virus fluorescence in DF-1 cells transfected with shDDX1-2 and infected with NDV-GFP (MOI = 0.1). **(D)** NSD14 NDV virus genome detected by qRT-PCR in the supernatant of chDDX1 knockdown (shDDX1-2) DF1 cells at 8, 16 and 24 hours after infection (MOI, 0.1 for 8, 16 and 24 hours). **(E)**–NSD14 NDV virus genome detected by qRT-PCR in chDDX1 knockdown DF1 cells (MOI, 0.1 for 8, 16 and 24 hours). **(F)** Luciferase assays for DF-1 cells cotransfected with RNAi plasmids (shDDX1-1, shDDX1-2, or shNC) and chIFN-β luciferase reporter plasmids and then stimulated with poly(I:C). **(G, H)** Luciferase reporter assays for DF-1 cells cotransfected with RNAi plasmids of chDDX1 (shDDX1-1, shDDX1-2, or shNC) and chIFN-β luciferase reporter plasmids and then infected with AIV or NDV. The difference between the experimental and control groups was *p < 0.05 or **p < 0.01.

To further investigate the role of chDDX1 in the chIFN-β signaling pathway in chicken cells, we constructed RNAi plasmids of chDDX1 (shDDX1-1, shDDX1-2, and shNC). To verify the specific knockdown target, the DF-1 cells were transfected with the shRNA plasmids and then transfected with expression plasmids 36 hours later. Twenty-four hours after that, the cells were harvested for western blot analysis. The results indicated that both shDDX1-1 and shDDX1-2 substantially reduced the expression of chDDX1. The results of the western blot and gray analyses are shown in **Figure 6A**. To verify whether knockdown of chDDXI increases virus replication, chDDX1 knockdown DF-1 cells were infected with VSV-GFP and NDV-GFP, and real-time fluorescence images were used to measure virus proliferation. The results showed that the virus replications of both VSV-GFP and NDV-GFP were faster in chDDX1 knockdown DF-1 cells than those in the control group (**Figures 6B, C**). Then, the chDDX1 knockdown (shDDX1-2) and normal DF-1 cells were infected with the NSD14 NDV and the viral RNAs inside the cells and in the supernatants were extracted separately for qPCR. The results showed that the virus copies both inside the cells (**Figure 6E**) and in the supernatants (**Figure 6D**) from chDDX1 knockdown cells were obviously higher than those in the control group. The chDDX1 knockdown cells transfected with luciferase reporter plasmids were then incubated with poly(I:C), AIV, or NDV, and luciferase reporter assays were performed. The results demonstrated that knocking down chDDX1 led to inhibited IFN-β promoter activation after the DF-1 cells were stimulated with poly(I:C) or infected with a virus (**Figures 6F–H**). These data showed that SH010 AIV, NSD14 NDV, and VSV-GFP induce chIFN-β *via* a chDDX1-dependent pathway and that chDDX1 may play an essential role in the antiviral innate immunity mediated by IFN-β in DF-1 cells.

### chDDX1 Activates IFN Signaling *via* the chIRF7 Pathway

**Figure 7:**
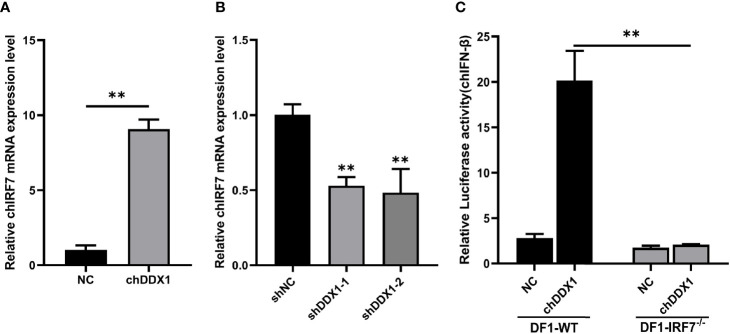
chDDX1 activated IFN signaling *via* the chIRF7 pathway. **(A)** Relative mRNA levels (qRT-PCR) of chIRF7 in DF-1 cells transfected with p3×FLAG-14-chDDX1 or p3×FLAG-14. **(B)** IRF7 expression (qRT-PCR) in DF-1 cells transfected with RNAi plasmids (shDDX1-1, shDDX1-2, or shNC). **(C)** chDDX1 expression in IRF7^-/-^ DF-1 cells (luciferase activity) in IRF7^-/-^ DF-1 cells and wild-type DF-1 cells transfected with p3×FLAG-14-chDDX1 or p3×FLAG-14 and IFN-β luciferase reporter plasmids. The difference between the experimental and control groups was *p < 0.05 or **p < 0.01.

To further explore the signal pathway through which chDDX1 activates IFN-β, DF-1 cells were transfected with a chDDX1 plasmid and an empty plasmid. Twenty-four hours after transfection, RNA was extracted for a qRT-PCR test, which showed that the transcription level of IRF7 was significantly upregulated (**Figure 7A**). After we endogenously knocked down the chDDX1, chIRF7 was significantly downregulated (**Figure 7B**). More critically, when chDDX1 was overexpressed in IRF7^-/-^ DF-1 cell lines, chIFN-β could not be activated (**Figure 7C**). Therefore, chIRF7 plays a critical role in the regulation of chIFN-β by chDDX1, and chDDX1 activates IFN signaling *via* the chIRF7 pathway.

### chDDX1 Interacts With Poly(I:C)

**Figure 8:**
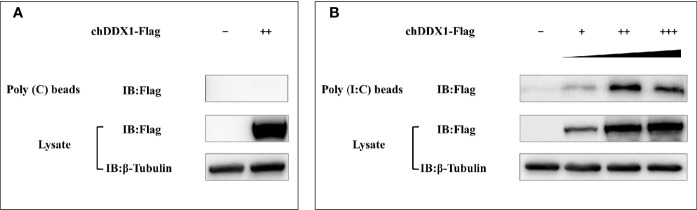
chDDX1 interacted with poly(I:C). **(A)** Pulldown assay (western blot) for the interaction of chDDX1 and poly(C) in DF-1 cells transfected with the indicated plasmids. **(B)** Pulldown assay (western blot) for the interaction of chDDX1 and poly(I:C).

Some members of the DExD/H-box helicases family have proven to be extremely important antiviral PRRs in mammals. It is unclear whether chDDX1 plays a similar function in innate immunity in chickens. *In-vitro* pulldown assays were used to explore the specific functions of chDDX1 in the IFN regulatory pathway. We found that chDDX1 bound to poly(I:C), a simulant of dsRNA, but not to poly(C), a simulant of ssRNA (**Figures 8A, B**). The *in-vitro* pulldown assay showed a strong and direct interaction between poly(I:C) and the chDDX1 protein, indicating that chDDX1 may act as an RNA PRR during IFN activation.

## Discussion

Chickens are the natural host of NDV and AIV. The discovery that the RIG-I gene, the primary RNA virus pattern recognition receptor (PRR) in mammals, is naturally absent in chickens has directed attention to studies of chicken RNA PRRs and their functions in antiviral immune responses. In some fish and mammals, DDX1 can regulate the expression of IFN-β, thereby playing a role in antiviral innate immunity and inhibiting virus replication (28, 40). However, DDX1 can also be hijacked by certain viruses to promote virus proliferation (30). The function of DDX1 in innate immunity in chickens is unclear.

For the host, upregulating the expression of certain immune-related genes is an important strategy in infection resistance. A qRT-PCR showed that chDDX1 mRNA was widely expressed in various tissues in healthy chickens (**Figure 3A**) and that chDDX1 was significantly upregulated in cells infected with AIV, NDV, or VSV-GFP (**Figures 3B–D**). Based on this finding, we speculated that chDDX1 may be involved in the antiviral immunity of chickens.

First, we cloned chDDX1 and analyzed the amino acid sequences of DDX1 from different animals. We found high conservation of the amino acid sequence of DDX1 between chickens and several mammals (**Figure 1A**). This result indicates that DDX1 may have a similar function in chickens and mammals. The predicted chDDX1 domain and homology modeling, as well as the phylogenetic analysis of DDX1 in different animals, including birds, fish, and mammals, are shown in **Figures 1B** and **2**. In cell experiments, we found that the overexpression of chDDX1 in CEFs or DF-1 cells strongly induces the expression of IFN-β, and this induction positively correlates with transfection time and the amount of chDDX1 plasmid transferred (**Figures 4A–C**). Besides IFN-β, other types of IFNs, including IFN-α and IFN-λ, were also activated by chDDX1 overexpression (**Figure 4D**). In addition, the overexpression of chDDX1 strongly upregulated the expression of IFN-stimulated genes, including PKR and Mx-1, as well as that of some pro-inflammatory cytokines, including IL-6 and IL-8 (**Figures 4E, F**). Virus infection experiments showed that VSV-GFP, NDV-GFP and NSD14 NDV replication were significantly inhibited in DF-1 cells overexpressing chDDX1 (**Figures 4G–J**). In summary, the overexpression of chDDX1 in DF-1 cells induced the expression of IFNs, IFN-stimulated genes, and proinflammatory cytokines and also inhibited VSV-GFP replication.

To identify the domain of chDDX1 that is indispensable to IFN induction, a series of truncated forms of chDDX1 were generated and their abilities to induce IFN-β promoter activity were compared (**Figure 5**). The results indicate that the first 250 amino acid sequences of chDDX1 contain the region that can activate IFN-β and that the region between 250 and 740 contains pivotal domains required for the induction of IFN-β promoter activity.

To further verify the immunomodulatory and antiviral activities of chDDX1, RNAi target sequences against chDDX1 (shDDX1-1, shDDX1-2) were designed. Both shDDX1-1 and shDDX1-2 substantially reduced chDDX1 expression (**Figure 6A**). To verify whether chDDX1 knockdown increases virus replication, chDDX1 knockdown DF-1 cells were infected with VSV-GFP, NDV-GFP and NSD14 NDV. The results showed that knocking down chDDX1 increased the replication of the above-mentioned viruses (**Figures 6B–E**). In addition, when DDX1 was knocked down by shRNA, the ability of the DF-1 cells to produce IFN-β in response to a viral infection or stimulation with poly(I:C) was significantly reduced (**Figures 6F–H**). These results further indicate that chDDX1 is pivotal to IFN-β regulation and to antiviral immunity.

These findings define chDDX1 as an IFN-β activator that plays a role in antiviral innate immunity. However, the specific role of DDX1 in antiviral innate immunity and the mechanism by which it activates IFN-β are still unclear. A qRT-PCR showed significantly upregulated IRF7 mRNA in cells that overexpressed chDDX1 (**Figure 7A**). After we knocked down endogenous chDDX1 in DF-1 cells using shRNA, the expression of IRF7 decreased significantly (**Figure 7B**). We then conducted a series of experiments using a chicken IRF7 knockout DF-1 cell line to demonstrate that chDDX1 activates IFN signaling *via* the chIRF7 pathway (**Figure 7C**). *In-vitro* pulldown assays showed that chDDX1 can bind to dsRNA mimics poly(I:C) but not to ssRNA mimics poly(C) (**Figures 8A, B**). This indicates that chDDX1 may be a dsRNA PRR that recognizes dsRNA virus or dsRNA intermediates produced during the replication of an ssRNA virus after the host has been infected, thus activating antiviral innate immunity. In short, IRF7 is a key protein in the antiviral innate immune function of chDDX1, and chDDX1 may act as a dsRNA sensor, relying on IRF7 to induce IFN-β and support antiviral innate immunity.

RIG-I is an important receptor in mammals; it can recognize dsRNA and plays a role in virus recognition and immune response regulation. However, RIG-I is absent from chicken innate immune systems (11). Our *in-vitro* pulldown assay showed a strong and direct interaction between poly(I:C) and the chDDX1 protein, indicating that chDDX1 may act as an RNA PRR during IFN activation. In addition, chDDX1 may be involved in innate immunity as a dsRNA sensor, thereby compensating for the absence of RIG-I. In summary, our results suggest that chDDX1 is an important IFN-β mediator and is involved in the RNA- and RNA virus-mediated chDDX1-IRF7-IFN-β signaling pathway. These results contribute to a more systematic understanding of the IFN-regulated signaling pathway and the innate immune system of chickens. They also provide reference data about the general and individual characteristics of the innate immunity of birds and mammals.

## Data Availability Statement

The original contributions presented in the study are included in the article. Further inquiries can be directed to the corresponding author.

## Ethics Statement

The protocols used in this study were approved by the Animal Research Ethics Committee of Shanghai Jiao Tong University (No. A2019008).

## Author Contributions

JS and YC designed the experiment. ZL performed the majority of the experiments. WZ, JW, and XY helped with the experiments. ZL and YC wrote the paper. ZW, JM, HW, and YY helped analyze the experimental results. All authors contributed to the article and approved the submitted version.

## Funding

This research was supported by the National Natural Science Foundation of China (31802175, 31872456, 32072864, and 32072865), the Shanghai Natural Science foundation (20ZR1425100), State Key Laboratory of Veterinary Biotechnology Foundation Grant (SKLVBF202107), and Startup Fund for Youngman Research at SJTU (SFYR at SJTU, 19X100040011).

## Conflict of Interest

The authors declare that the research was conducted in the absence of any commercial or financial relationships that could be construed as a potential conflict of interest.

## Publisher’s Note

All claims expressed in this article are solely those of the authors and do not necessarily represent those of their affiliated organizations, or those of the publisher, the editors and the reviewers. Any product that may be evaluated in this article, or claim that may be made by its manufacturer, is not guaranteed or endorsed by the publisher.
